# Structural Characterization of an LPA_1_ Second Extracellular Loop Mimetic with a Self-Assembling Coiled-Coil Folding Constraint

**DOI:** 10.3390/ijms14022788

**Published:** 2013-01-29

**Authors:** John K. Young, Benjamin T. Clayton, Alexandra Kikonyogo, Truc-Chi T. Pham, Abby L. Parrill

**Affiliations:** 1 Department of Chemistry and Computational Research on Materials Institute, The University of Memphis, Memphis, TN 38152-3550, USA; E-Mails: benjaminclayton@Upike.edu (B.T.C.); mkknyogo@memphis.edu (A.K.); tcpham@memphis.edu (T.-C.T.P.); 2 Department of Chemistry, Christian Brothers University, Memphis, TN 38104, USA; E-Mail: jyoung7@cbu.edu (J.K.Y.)

**Keywords:** G protein-coupled receptor, GPCR, lysophosphatidic acid, LPA, NMR, GPCR segment model

## Abstract

G protein-coupled receptor (GPCR) structures are of interest as a means to understand biological signal transduction and as tools for therapeutic discovery. The growing number of GPCR crystal structures demonstrates that the extracellular loops (EL) connecting the membrane-spanning helices show tremendous structural variability relative to the more structurally-conserved seven transmembrane α-helical domains. The EL of the LPA_1_ receptor have not yet been conclusively resolved, and bear limited sequence identity to known structures. This study involved development of a peptide to characterize the intrinsic structure of the LPA_1_ GPCR second EL. The loop was embedded between two helices that assemble into a coiled-coil, which served as a receptor-mimetic folding constraint (LPA_1_-CC-EL2 peptide). The ensemble of structures from multi-dimensional NMR experiments demonstrated that a robust coiled-coil formed without noticeable deformation due to the EL2 sequence. In contrast, the EL2 sequence showed well-defined structure only near its *C*-terminal residues. The NMR ensemble was combined with a computational model of the LPA_1_ receptor that had previously been validated. The resulting hybrid models were evaluated using docking. Nine different hybrid models interacted with LPA 18:1 as expected, based on prior mutagenesis studies, and one was additionally consistent with antagonist affinity trends.

## 1. Introduction

G protein-coupled receptors (GPCRs) are a large family of integral membrane proteins that play critical roles in cellular signaling. Members of this family are common drug targets, estimated to be the target of up to 50% of drugs [1,2]. Family members not currently targeted by existing drugs are the subject of extensive drug discovery research programs. The lysophosphatidic acid (LPA) receptors are among this latter group of GPCR due to their roles in cellular survival, migration, proliferation, and apoptosis [3–5]. These LPA_1_ receptor-mediated actions have been linked to the survival and metastasis of several cancer cell types [6–8]. This therapeutic relevance of LPA_1_ stimulated our development and validation of an LPA_1_ receptor model to guide the identification of LPA_1_ receptor antagonists [9–13].

**Figure 1:**
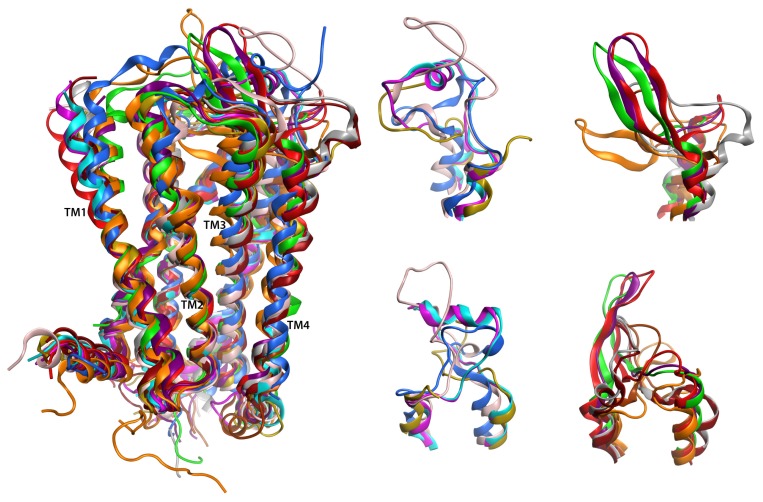
Superposition of twelve crystallized GPCR family members. Rhodopsin (1F88 [21], orange), β2-adrenoceptor (2RH1 [19], magenta), β1-adrenoceptor (2VT4 [18], cyan), adenosine A2a (3EML [17], pink), dopamine D3 (3PBL [15], brown), chemokine CXCR4 (3Oe0 [16], green), histamine H1 (3RZE [14], mustard), muscarinic acetylcholine M2 (3UON [22], brick red), muscarinic acetylcholine M3 (4DAJ [33], grey), κ-opioid (4DJH [23], red), μ-opioid (4DKL [24], purple), and S1P_1_ (3V2Y [25], blue) are shown using ribbon representations. **Left**: Complete backbone rendered as ribbon with the exception of T4 lysozyme replacements for IL3. The four TM segments closest to the viewer are labeled. **Right**: Two views of EL2 and extracellular ends of surrounding helices with structures separated into two groups for clarity.

Substantial interest in the structures of GPCR to accelerate drug candidate discovery has so far led to over a dozen different nearly-complete class A (rhodopsin-like) GPCR with published crystallographic [14–27] or solid-state NMR [28,29] structures (some in multiple states or with a variety of ligands, rhodopsin alone is represented in approximately 20 different PDB entries as reviewed in [30]), and numerous structures of the *N*-terminal domain of class B (secretin family) and C (metabotropic glutamate/pheromone) GPCR family members (reviewed in [30]). Figure 1 demonstrates that representative crystallized class A GPCR structures show strong structural conservation from the intracellular end of the helical bundle up toward the last third of the extracellular end of the helical bundle. Extensive structural diversity is displayed among the loops exposed to the extracellular space, particularly the longer second (EL2, highlighted in Figure 1) and third (EL3) extracellular loops. Among the class A GPCR family members shown, only three EL2 architectures are repeated and six additional architectures are each present in only one family member. One repeated architecture is exhibited by the β1 and β2 adrenoceptors. A second is common to the M2 and M3 muscarinic acetylcholine receptors. The third is shared by the CXCR4 chemokine receptor and the opioid receptors (two of four opioid receptors shown in Figure 1). Overall sequence identity between the receptors with common structure is 55% or higher. This superposition indicates that comparative models of GPCR, such as our model of LPA_1_, will be highly accurate at the intracellular half of the helical bundle, moderately accurate at the extracellular half of the helical bundle, and poorly accurate in the loop segments connecting the transmembrane segments, unless a close homolog exhibiting a shared architecture is known. Experimental characterization of the highly variable connecting loop segments is, therefore, of value to improve the quality of comparative models and provide structural insights into the source of ligand selectivity.

Numerous structural characterization studies on sequence fragments from GPCR have been performed (reviewed in [30]). These studies indicate that carefully engineered designs including disulfide bonds or self-assembling coiled-coils can be used to promote folded structures, in contrast to isolated loop fragment sequences which are generally relatively extended and flexible. We previously reported the structure of the S1P_4_ first extracellular loop (S1P_4_-EL1) using an antiparallel coiled-coil and disulfide bond to provide a folding constraint [31]. This design required 20% trifluorethanol to promote helicity due to the relatively short coiled-coil sequences used. Nevertheless, this prior design led to the identification of a short 3^10^ helical segment in the S1P_4_ first extracellular loop, and demonstrated the ability of the peptide to selectively recognize a truncated version of the natural agonist (phosphoethanolamine) but not the truncated version of a related non-agonist lipid (*N*-acylethanolamine). The relevance of this structural feature in the full-length receptor is supported by the recent crystallographic structure of the closely-related S1P_1_ receptor, which shows a short helix in the corresponding location. In the current study, we report on the structure of the substantially longer LPA_1_ second extracellular loop sequence in aqueous buffer using a longer optimized antiparallel coiled-coil [32] to promote self-assembly in the absence of both trifluoroethanol and an interhelical disulfide bond. This improved second-generation design was applied to characterize the intrinsic structure of the LPA_1_ second extracellular loop sequence in aqueous solution, demonstrating that this loop contains no intrinsic secondary structure elements, but does exhibit a strong outward bend at the *C*-terminal end leading into the *C*-terminal helix.

## 2. Results and Discussion

### 2.1. Peptide Design

**Figure 2:**
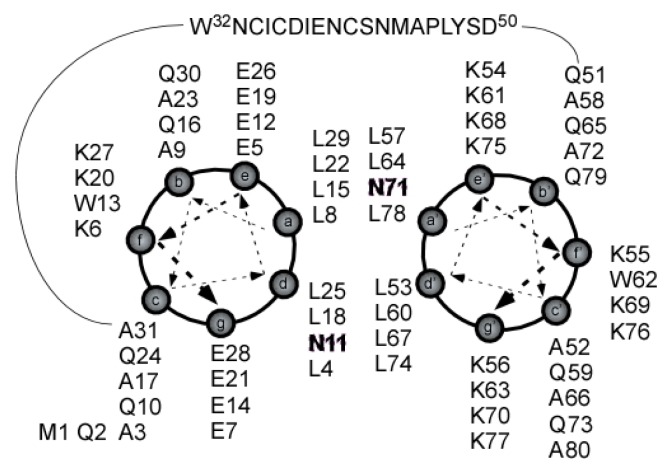
Sequence of LPA_1_-CC-EL2 shown with helical wheels for the coiled-coil segment. N11 and N71 have been highlighted to show that they should occur in cross positions.

In order to investigate loop conformations, a coiled-coil scaffold was constructed to provide a folding constraint for the particular loop of interest. Oakley *et al.* have previously studied designed peptide sequences for their ability form parallel and anti-parallel coiled-coils [32]. The ability of peptide pairs to form a coiled-coil was confirmed using CD. The placement of two Asn residues was important in distinguishing a parallel versus an anti-parallel coiled-coil. In this study we used their sequences along with EL2 of LPA_1_ in a continuous sequence (LPA_1_-CC-EL2) which formed an anti-parallel coiled-coil due to the placement of two Asn residues in positions a′ and d (Figure 2). EL2 was chosen for this study since it has a wide variety of structures across the different GPCR (Figure 1).

### 2.2. Circular Dichroism (CD)

**Figure 3:**
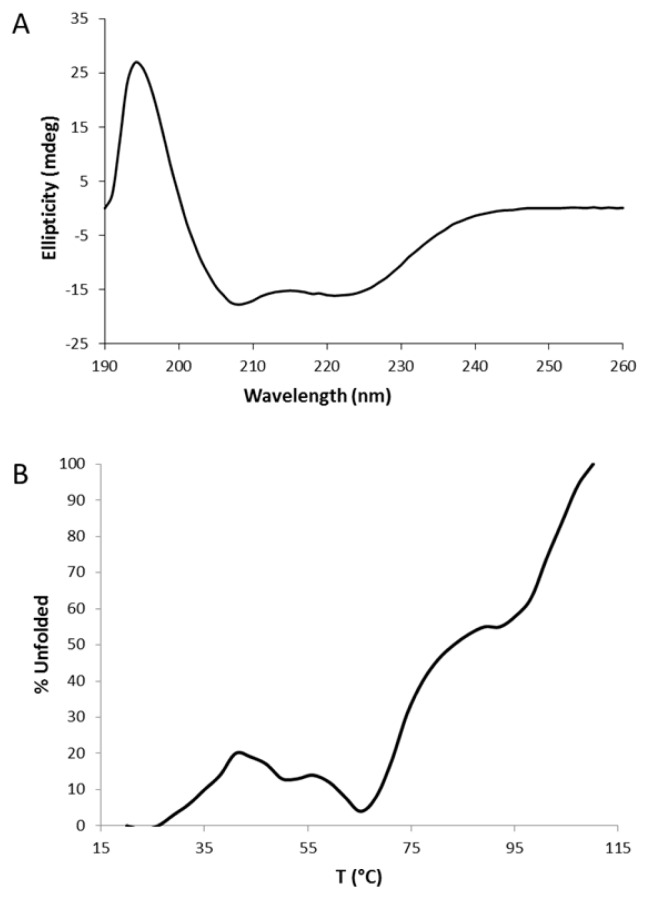
LPA_1_-CC-EL2 CD spectra as a function of (**A**) wavelength and (**B**) temperature.

**Figure 4:**
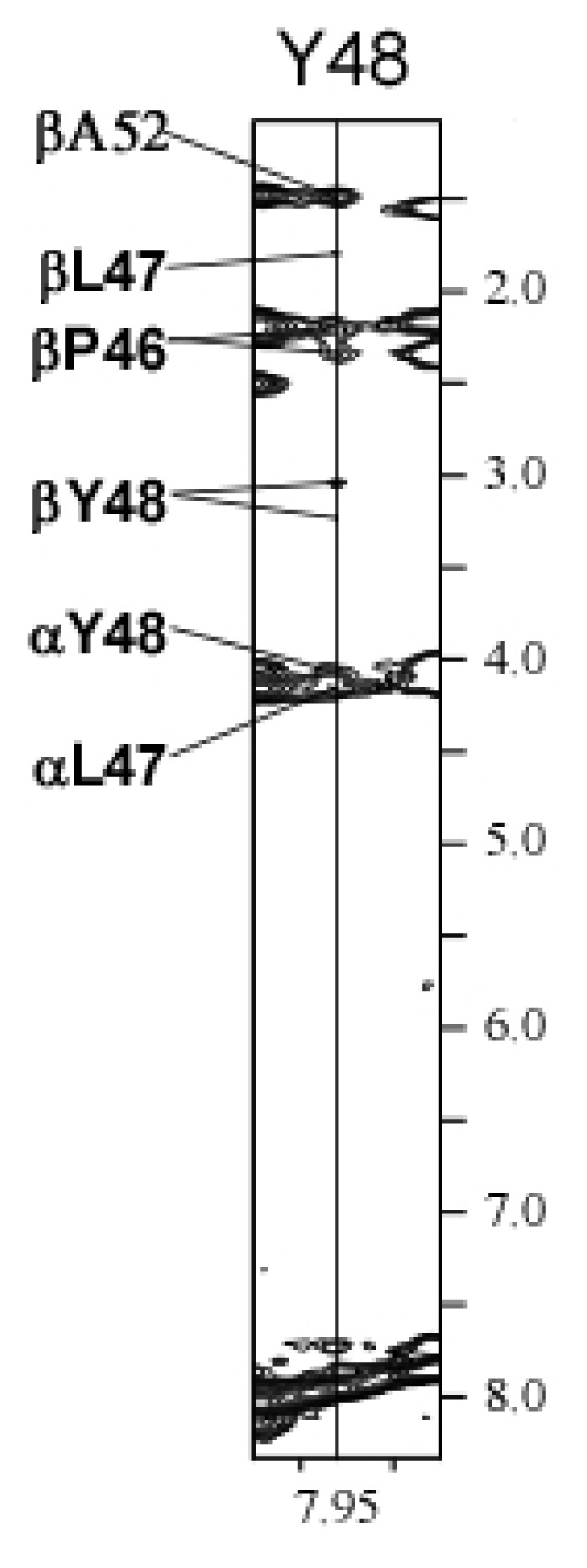
^15^N-NOE contacts of Y48 with P46, L47, Y48, and A52 that define the outward bend at the *C*-terminal end of LPA_1_ EL2.

Circular dichroism spectra were collected in order to define an appropriate buffer for NMR studies, in which the LPA_1_-CC-EL2 peptide exhibited the predominantly helical structure expected on the basis of the design. Once an appropriate buffer was selected, thermal denaturation was performed using 220 nm to follow denaturation of the α-helical secondary structure. Figure 4 shows a wavelength scan at 20 °C as well as thermal denaturation. Deconvolution of the secondary structure using the CDSSTR algorithm with reference set 7 [34] on Dichroweb [35–37] indicates the LPA_1_-CC-EL2 peptide is 65% helical, 11% strand, 9% turns, and 14% disordered. Thermal denaturation data demonstrated that LPA_1_-CC-EL2 unfolding was not completely cooperative, as a plateau was apparent at about 90 °C. The CD thermal denaturation of the LPA_1_-CC-EL2 peptide (Figure 3) is consistent with the thermal denaturation studies performed by Oakley *et al.* [32] on the separate coiled-coil sequences as an equimolar mixture, which showed a single thermal denaturation below 80 °C (the highest temperature tested).

### 2.3. Chemical Shift Assignments and Helical Calculations

Sequence-specific chemical shift assignments for LPA_1_-CC-EL2 were obtained from the interactive use of the backbone 3D NMR experiments listed in experimental methods. These chemical shifts were then used to obtain the sidechain assignments (aromatics unassigned). Chemical shifts have been deposited in the BioMagResBank under accession number 17993. The backbone nitrogen of E26 has gone unassigned; however, its sidechain chemical shifts were obtained using back correlations from K27. The TALOS program provided 119 Φ and Ψ dihedral angles from the chemical shift data. A total of 715 ^1^H–^1^H distance restraints (NOEs) from the 3D ^15^N-NOESY-HSQC and ^13^C-NOESY-HSQC experiments were employed in the CNS structure calculation. Four NOE peak intensity categories were used to classify intra ^1^H–^1^H upper distance limits; strong (<2.7 Å), medium (<3.3 Å), weak (<4.0 Å), and very weak (<4.5 Å). These two types of constraints were used in the initial CNS structure calculations. Once a calculation was completed, the NOEs and dihedrals were assessed based on violations. If a distance or dihedral was violated, it was redefined to the next weakest category for NOEs or 5° was added to the dihedral range. This continued until there were no NOE violations greater than 0.5 Å and no dihedral violated by greater than 10°. At this point another calculation was performed that defined 24 hydrogen bonds down the two helices to further stabilize their structures.

### 2.4. Coiled Coil

Initial structure calculations employed short and medium range NOEs to refine the two separate helical structures. Both TALOS and chemical shift analysis (data not shown) indicated helical structure from residues 3–31 and 50–78. Once these structures converged, the coiled-coil was initially defined by 10 distances observed in the ^13^C-NOESY-HSQC data. Specifically, very weak distances were defined between the methyl groups of L4 to L74, L18 to L60, L22 to L64, L25 to L53 and L29 to L57 (two distances for each pair). These distances represented the only unique chemical shift pairs in the predicted coiled-coil since most of the sidechain leucine interactions were overlapped. However, this proved to be sufficient to define the coiled-coil structure. One key feature in the design of this coiled-coil was the presence of N11 and N71. The positions of these residues are important to promote an anti-parallel coiled-coil. There were two NOEs observed in the ^15^N-NOESY-HSQC, one from the NH_2_ group of N11 to the methyl of L74 and the other from the NH_2_ group of N11 to the NH_2_ group of N71. First, a calculation was performed that defined the distance for N11 to L74. In all the resulting structures from previous coiled-coil calculations the N11 and N71 residues were at positions a′ and d which indicated an anti-parallel coiled-coil. Also the sidechain NH_2_ group of N11 pointed to the sidechain O of N71 which still placed the two NH_2_ groups close enough to show a weak NOE, therefore another calculation contained this distance restraint.

### 2.5. Loop

**Figure 5:**
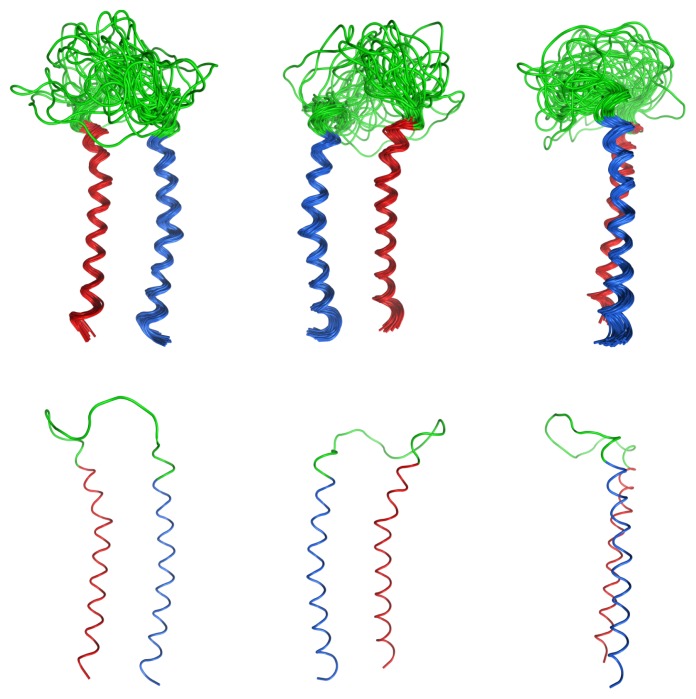
Superimposition of 36 representative low energy structures from the CNS calculation. **Left**: *N*-terminal helix on the left and *C*-terminal helix on the right, **Center**: *N*-terminal helix on the right and *C*-terminal helix on the left, and **Right**: side view with the *C*-terminal helix in front. A single representative structure has also been shown for a simplified view.

The last few rounds of calculations employed short and medium range distance restraints in the loop. Most of the loop residues did not result in any NOEs in either the ^15^N-NOESY-HSQC or ^13^C-NOESY-HSQC suggesting a complete lack of structure in these residues of the loop. However, there were short range (*i* to *i* and *i* to *i* ± 1) ^15^N-NOEs observed from residues Ala45 through Ala52. There were also three medium range ^15^N-NOEs involving Pro46 through Ala52 (Figure 4). The last calculation included these NOEs which produced an interesting conformation in this part of the loop (see Section 2.6). The final round of structure calculations consisted of 100 structures. Table 1 shows statistical information used for and resulting from the final calculation of the 58 lowest energy structures out of the 100 used in the final round of structure calculations. Of these 58 lowest energy structures, 36 representatives for the LPA_1_-CC-EL2 were superimposed as shown in Figure 5.

### 2.6. Overall Structure

The overall structure of LPA_1_-CC-EL2 does not converge to a single family (backbone RMSD 4.01 Å) due to a lack of NOEs in the loop. However, each helix does show convergence (backbone RMSD 0.63 Å and 0.58 Å respectively) as does the coiled-coil (backbone RMSD 0.95 Å). The RMSD values for the bonds, angles, impropers, NOEs, and dihedrals are acceptably low (<3% of values for bonds, angles, impropers and NOEs, ~20% of value on dihedrals). Overall, >80% of the residues of the LPA_1_-CC-EL2 family fall in favored or allowed regions of the Ramachandran plot indicating a well calculated structure. The set of 36 structures in Figure 5 were submitted to the Research Collaboratory for Structural Bioinformatics (RCSB) Protein Data Bank (PDB) with the RCSB ID code RCSB102692 and PDB ID code 2LQ4.

**Figure 6:**
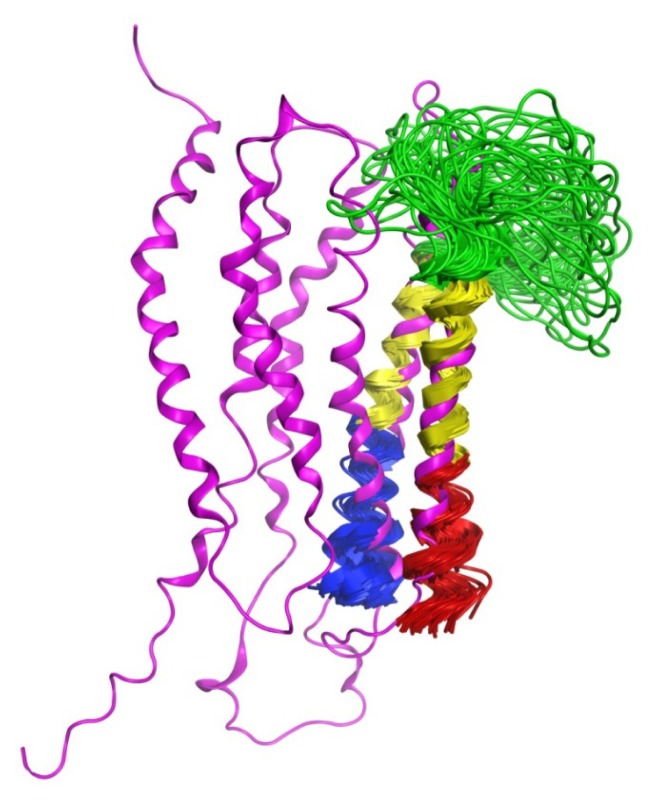
Superimposition of the calculated structures on helix 4 and 5 of the validated LPA_1_ receptor model. The LPA_1_ receptor model is shown as a magenta ribbon with TM1 on the left side, the *N*-terminal ends of the LPA_1_-CC-EL2 peptide structures are shown as red ribbons (superposed on TM4 of the LPA_1_ model), the *C*-terminal ends as blue ribbons (superposed on TM5 of the LPA_1_ model), and segments superposed on the LPA_1_ receptor model are shown as yellow ribbons.

**Figure 7:**
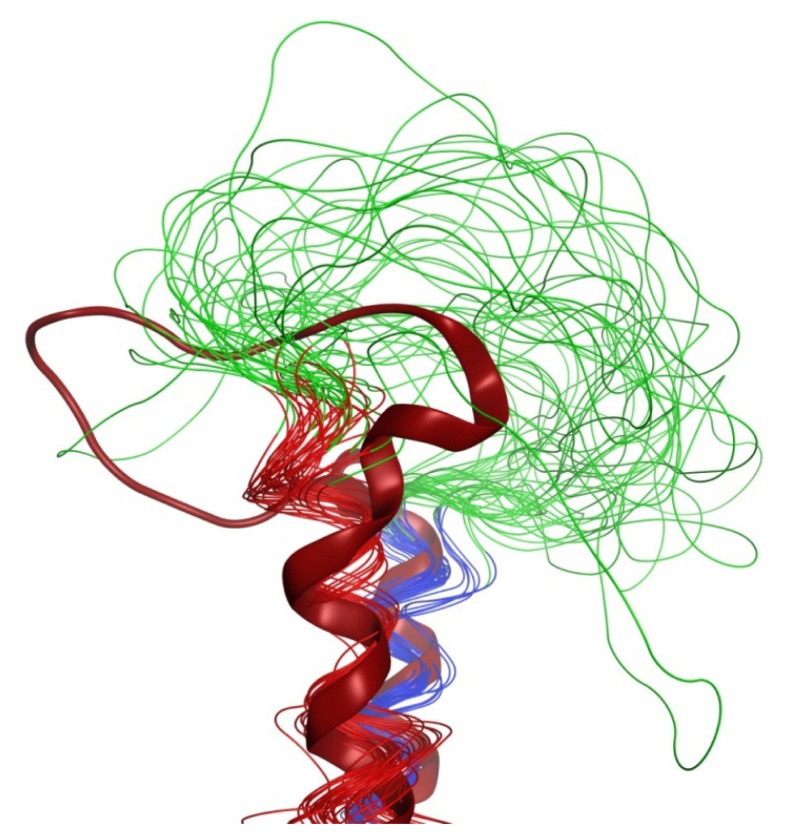
Comparison of outward bends in LPA_1_-CC-EL2 NMR structures (*N*-terminal helix: red, *C*-terminal helix: blue, EL2: green) and EL2 from the M2 muscarinic acetylcholine receptor crystal structure (3UON [22], brick red).

The results of these calculations as well as the CD wavelength spectra and thermal denaturation show that the peptide sequences from Oakley *et al.* [32] do indeed form a coiled-coil when connected by a linking peptide. These sequences, therefore, provide an excellent scaffold within which loop conformations can be investigated. These results show that the EL2 conformation of the LPA_1_ receptor does not contain any well-defined secondary structure, analogous to EL2 in six of the class A GPCR members for which crystal structures are available (Figure 1). However, there was an interesting result in the conformation around residues P46 to A52, due to NOE contacts from the sidechains of residues P46 (β-hydrogen), Y48 (δ- and ɛ- hydrogens), and A52 (β-hydrogen) to the backbone amide hydrogens of residues L47, Y48, and D50 (Figure 4). Although the sidechain of coiled-coil residue A52 is involved in this network of contacts, the β-hydrogens of the tyrosine residue that occurs in full-length LPA_1_ could make the same contacts, thus this conformation appears to be an intrinsic property of the LPA_1_ EL2 loop sequence. Due to these NOE contacts, this part of the loop was always in a bent conformation (Figure 5); however it did not converge into a single conformation suggesting that this part of the loop is limited to a small amount of conformational space while the rest of the loop has a greater range of conformational freedom. This would represent the carboxy-terminal end of the loop transitioning into helix 5 of the LPA_1_ receptor. The resulting structures from these calculations were superimposed into the corresponding helix4–EL2–helix5 of our LPA_1_ receptor model in order to illustrate how this bend in EL2 fit onto the overall structure (Figure 6) and to provide starting structures for hybrid LPA_1_ receptor model development. Of these 58 structures, only 2 showed any overlap of the loop with atoms of the receptor. The bend in the loop places the loop in an outward conformation that leaves the top of the receptor open, potentially to accommodate a structured segment within the *N*-terminus as observed in the recently characterized S1P_1_ receptor [25]. The outward bend of EL2 is also observed in the recently characterized M2 muscarinic acetylcholine receptor although at the amino-terminal end of EL2 rather than the carboxy-terminal end (Figure 7).

### 2.7. Hybrid LPA_1_ Receptor Models

**Figure 8:**
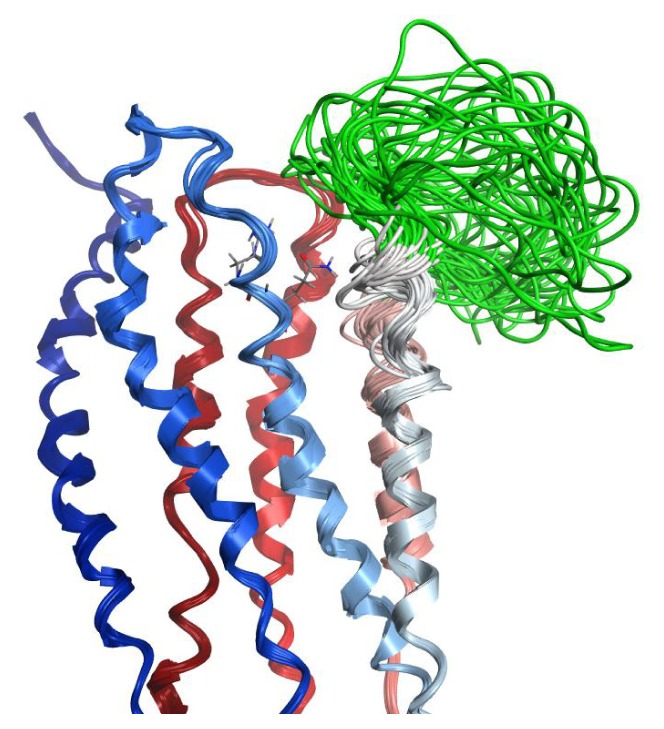
Hybrid NMR/Model LPA_1_ Receptor Structures. Receptor backbone from the previously validated LPA_1_ receptor model is rendered as a ribbon colored from blue at the amino terminus to red at the carboxy terminus. EL2 segment structures modeled based on the NMR structures of LPA_1_-CC-EL2 are colored green. R124^3.28^ and Q125^3.29^ from one model are shown as sticks for clarity (all-atom RMSD for atoms in these amino acids in the 40 models is 0.9 Å).

The 58 lowest-energy LPA_1_-CC-EL2 NMR structures were each superposed on the validated LPA_1_ receptor model in order to produce a hybrid NMR/model structure by multi-template homology modeling (Figure 6). The EL1 portion of the LPA_1_ receptor model had previously been remodeled on the basis of the NMR structure of our shorter coiled-coil EL1 mimetic of the S1P_4_ receptor [31], which exhibits a short 3^10^ helical turn at the same location as a short helix noted in the recent S1P_1_ crystal structure. Thus the 58 resulting models are hybrids of the NMR structure data for LPA_1_-CC-EL2, S1P_4_-EL1, and the original template LPA_1_ receptor model. One of the resulting hybrid LPA_1_ receptor model structures showed EL2 wrapped around EL3 and was eliminated from further consideration. Seventeen additional structures showed very close contacts (<1.6 Å) between atoms in the EL2 structure and residues R124^3.28^ or Q125^3.29^ at the extracellular end of TM3 (Figure 8), which have been demonstrated by previous modeling and mutagenesis to form required interactions with the natural agonist, LPA. These close interactions would prevent LPA interaction at these sites, so these 17 models were also eliminated from further consideration. The remaining 40 models are shown in Figure 8.

### 2.8. Docking Results

**Figure 9:**
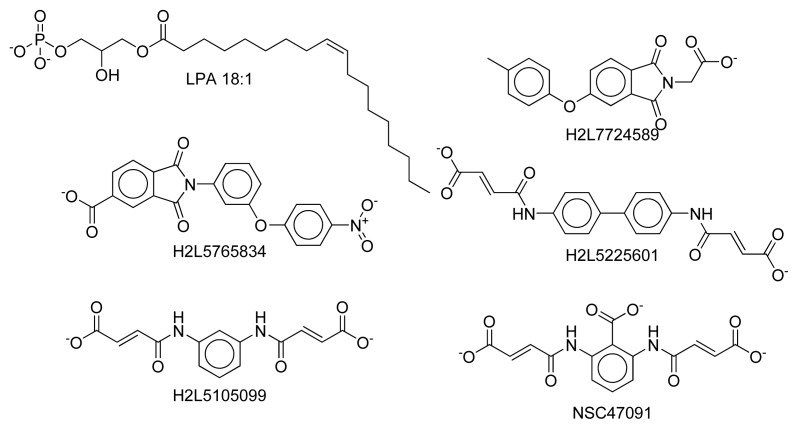
Chemical structures used in docking studies.

**Figure 10:**
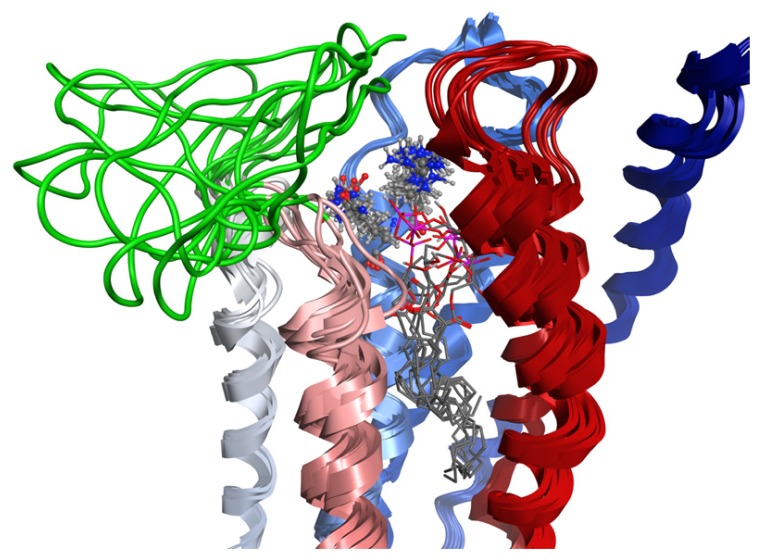
Hybrid LPA_1_ model complexes with LPA (stick) headgroup placed near R124^3.28^ and Q125^3.29^ (ball & stick). EL2 segment shown as green ribbons, remainder of LPA1 colored from blue at the amino terminus to red at the carboxy terminus.

**Figure 11:**
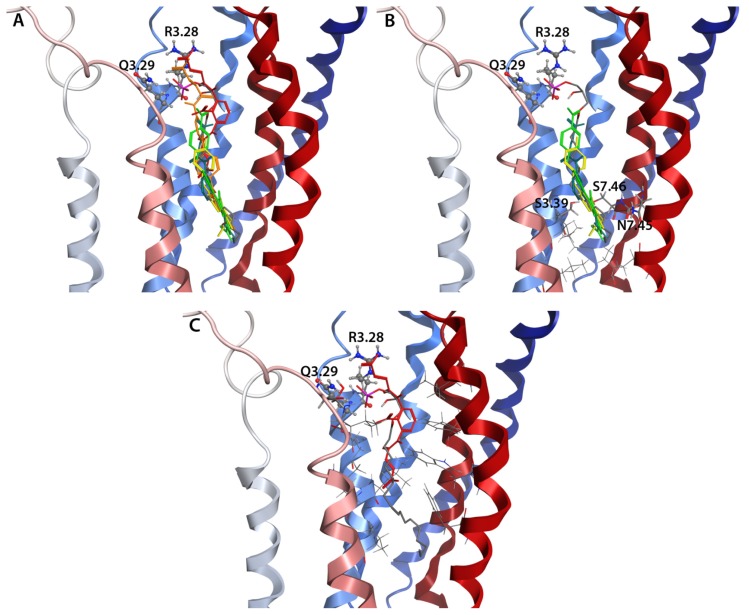
Consistency of LPA_1_ hybrid model generated from chain 3 of 2LQ4 with pharmacological trends. LPA_1_ is shown as ribbons shading from blue at the amino terminus to red at the carboxy terminus in all panels. R3.28 and Q3.29 are shown as ball&stick models in all panels. Antagonists are colored according to potency, with the most potent antagonist H2L5765834 colored green, followed by H2L5105099 (blue-green), H2L7724589 (yellow), H2L5226501 (orange), and the inactive compound NSC47091 (red). LPA is shown using element colors in all panels. (**A**) Superposition of all docked ligands; (**B**) Superposition of three most active antagonists showing residues within 4.5 Å of the buried carboxylate groups as either lines (hydrophobic sidechains) or labeled sticks (polar sidechains); (**C**) Docked pose of inactive compound showing residues within 4.5 Å of the buried carboxylate groups as either lines (hydrophobic sidechains) or labeled sticks (polar sidechains).

Docking studies were performed for the ligands shown in Figure 9 using each of the 40 hybrid LPA_1_ receptor models from Figure 8 as docking targets. These studies were performed in order to determine if specific members of the NMR ensemble were compatible with known features of the full-length LPA_1_ receptor. LPA 18:1 is expected to interact with residues R124^3.28^ and Q125^3.29^ at the extracellular end of TM3, as mutations of either of these residues to alanine produces receptors that are either only weakly activated or are not activated by LPA 18:1 [12]. The corresponding sites in the closely-related S1P_1_ receptor, R120^3.28^ and E121^3.29^, have been demonstrated to interact with the phosphate and ammonium groups of a co-crystallized antagonist [25]. Thirty-one models were eliminated from further consideration due to high-ranking poses of LPA with phosphate groups placed in the lower end of the binding pocket, distant from both R124^3.28^ and Q125^3.29^. Such a result suggests that hybridization of that particular loop geometry has altered the headgroup recognition pocket, and therefore the resulting hybrid does not represent a biologically meaningful model of the full-length receptor. The remaining nine models in which the top-ranked LPA pose exhibited a headgroup position near R124^3.28^ and Q125^3.29^ are shown in Figure 10. Figure 11 illustrates the consistency of complexes docked into the hybrid model generated from chain 3 in PDB entry 2LQ4 with the known antagonist pharmacology. The active antagonists, while all having a buried carboxylate functional group, have that polar functional group in a relatively polar subpocket formed by S3.39, S7.46, and N7.45 (panel B). In contrast, the inactive compound, NSC 47091, buries two carboxylic acid functional groups in relatively hydrophobic pockets, including S5.37 as the only polar sidechain (panel C).

### 2.9. Full-length Sequence Context

**Figure 12:**
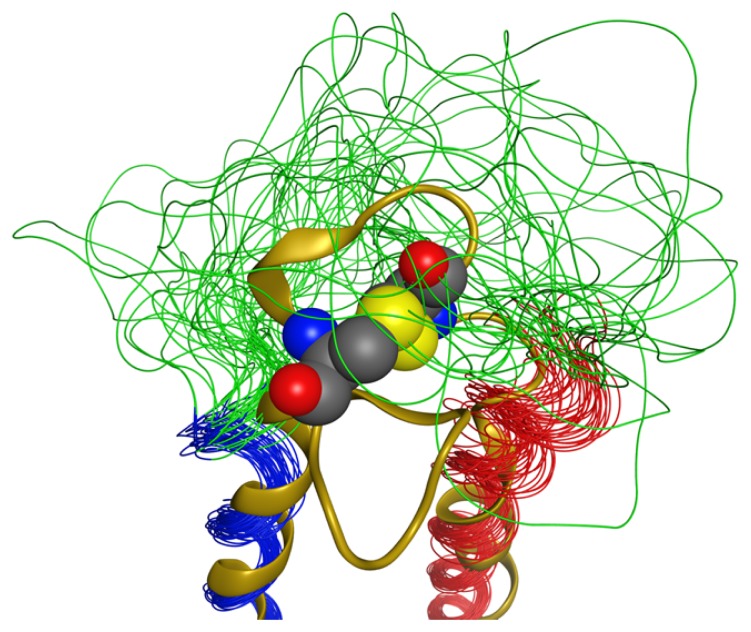
Comparison of LPA_1_-CC-EL2 ensemble (*N*-terminal helix: red, *C*-terminal helix: blue, EL2: green) to the S1P_1_ EL2 (3V2Y [25], yellow). The disulfide bonded cysteine residues in the S1P_1_ EL2 are shown as spacefilling models.

The present study demonstrates that the sequence found in the LPA_1_ EL2 is largely flexible and unstructured, even when the termini are held close together by a coiled-coil scaffold. Two characteristics that might reduce the conformational space available to this loop in the full-length receptor are disulfide bonds and contacts with other extracellular segments including EL1, EL3, or the amino terminus. The recently crystallized S1P_1_ structure has an EL2 identical in length which exhibits 48% residue identity and 57% homology to the LPA_1_ EL2, and is the only class A GPCR crystallized to date that lacks an interloop disulfide bond between cysteine residues in EL1 and EL2. S1P_1_ instead exhibits an intraloop disulfide bond between two cysteine residues in EL2 and a second intraloop disulfide bond between two cysteine residues in EL3. A superposition of the NMR ensemble on the S1P_1_ EL2 is shown in Figure 12. Like S1P_1_, the LPA_1_ receptor lacks cysteine in EL1, and exhibits conservation of the cysteine residues involved in the internal EL2 disulfide bond (C184 and C191 in LPA_1_) [38]. However, LPA_1_ additionally contains a third cysteine residue in both EL2 (C186) and EL3 that is not found in S1P_1_. The additional cysteine residues prevent direct inference of matching disulfide bonds in S1P_1_ and LPA_1_. However, it is likely that one or more of the three LPA_1_ EL2 cysteine residues participates in intraloop or interloop disulfide bonds in the context of the full-length receptor that would provide an additional conformational restriction relative to the LPA_1_-CC-EL2 peptide characterized with cysteine residues in reduced form. However, it is not clear at this time if the LPA_1_ EL2 structure in the context of the full-length LPA_1_ receptor should closely match that of S1P_1_ due to the differences in number of cysteine residues noted. The NMR ensemble of LPA1-CC-EL2 showed no consistent Cys–Cys close contacts that suggest an intrinsic disulfide pairing preference.

## 3. Experimental Section

### 3.1. Sample Preparation

The second extracellular loop of LPA_1_ was inserted between helical segments known to form an antiparallel coiled-coil to form a synthetic protein sequence, termed LPA_1_-CC-EL2. LPA_1_-CC-EL2 DNA was prepared by PCR using 7 overlapping oligonucleotides and two terminal primers, and incorporated into pET-32 Ek/LIC (EMD Millipore) using ligation-independent cloning to produce the plasmid containing a hexahistidine tag. The resulting expression vector was confirmed with DNA sequencing (Molecular Resource Center, UTHSC, Memphis, TN) and then transformed into *E. coli* BL21(DE3) cells for protein expression. Cell cultures were grown in minimal media containing 1 L of _13_C_6_-glucose as the sole source of ^13^C and 1.0 g/L of ^15^NH_4_Cl as the sole source of ^15^N. This growth was performed as previously described, with no alterations [31]. Cells were grown at 37 °C and protein expression induced with 0.1 mM IPTG at an OD_600_ of 0.6. The cells were pelleted by centrifugation and stored at −80°C. The cell pellet was lysed using the CelLytic B Plus Kit (Sigma-Aldrich, MI, USA) and loaded directly onto a Ni-NTA affinity column (GE Healthcare, New Jersey, NJ, USA), washed with 40 mM imidazole in 20 mM sodium phosphate buffer, pH 7.4, containing 500 mM sodium chloride followed by elution with 500 mM imidazole in the same buffer. The desired peptide (LPA_1_-CC-EL2) was then cleaved from the His-tag using recombinant enterokinase (EMD BioSciences) and purified by HPLC using a reverse-phase C18 preparative column. Anion-exchange chromatography was then used to separate the desired protein from nonspecific cleavage products, followed by confirmation using ESI-mass spectroscopy and sodium dodecyl sulfate polyacrylamide gel electrophoresis (SDS-PAGE). The final protein sample was concentrated to either 0.1 mM (for CD thermal denaturation) or 0.5 mM (for NMR studies), and the solvent exchanged to 50 mM Tris-HCl, 1.0 mM DTT, 1 mM sodium azide, pH 6.0 and 10% D_2_O.

### 3.2. Circular Dichroism (CD) Data

All CD data were acquired on an Aviv 410 spectropolarimeter using a 1 mm cell. Wavelength scans were collected for 0.1 mg/mL samples at 1 nm increments over a wavelength range of 190–260 nm at room temperature. Thermal denaturation experiments were performed over a temperature range from 20 °C to 110 °C at a wavelength of 220 nm. Ellipticities (φ) measured during thermal denaturation were converted to % unfolded at each temperature (*T*) based on the assumption that the structure was fully folded at the lowest temperature (*T*min) and fully unfolded at the highest temperature (*T*max) using Equation 1 [39]:

(1) $$\%\;\text{unfolded}=\left(1-\frac{\varphi^{T}-\varphi^{T\text{max}}}{\varphi^{T\text{min}}-\varphi^{T\text{max}}}\right)\times 100$$

### 3.3. NMR Data

All NMR data were acquired at 25 °C on a Varian VNMRS 500 MHz spectrometer using a 5 mm triple-resonance ^1^H, ^13^C, ^15^N probe with *z*-axis gradients. The sequential backbone assignments were accomplished through the interpretation of the experiments: HNCA, HN(CO)CA, HNCACB, CBCANH, HNCO, HN(CA)CO and ^15^N-NOESY-HSQC. Sidechain assignments were obtained through the H(CCO)NH-TOCSY, (H)C(CO)NH-TOCSY and HCCH-TOCSY experiments. NOE data was interpreted from the ^15^N-NOESY-HSQC and ^13^C-NOESY-HSQC experiments, both with a 150 ms mixing time. Data was processed using NMRPipe [40] software and analyzed with PIPP [41].

### 3.4. Structure Calculations

NMR chemical shift values for ^15^N, ^1^H^α^, ^13^C^α^, ^13^C^β^, and ^13^C′ were used in the TALOS program [42] for predictions of Φ and Ψ dihedral angles. NOE distances were obtained from the 3D ^15^N-NOESY-HSQC and ^13^C-NOESY-HSQC experiments. Dihedral angle and NOE restraints were used as input for the structure calculation with the crystallography and NMR system (CNS version 1.1) program [43]. A series of structure calculations ended when a family of structures was obtained in which no NOE distance was violated by >0.5 Å, no dihedral angle was violated by >10°, and low energies and good convergence were obtained.

### 3.5. Hybrid Receptor Model Development

Hybrid LPA_1_ receptor models were developed using a previously validated model of the LPA_1_ receptor [9,12,44] and the NMR structures of LPA_1_-CC-EL2 as input for multi-template homology modeling by the MOE software [45]. The hybrid models were generated using residues Leu29-Gln51 from the NMR structures of LPA_1_-CC-EL2 as the basis for modeling residues Ser183-Ser205 and the previously published LPA_1_ receptor model as the basis for modeling the remainder of the structure. The resulting models did not include residues 1–35 and 332–364 (*N*- and *C*-termini).

### 3.6. Docking

LPA 18:1 and several compounds exhibiting varying potencies as LPA receptor antagonists (H2L5765834 K_i_ = 48 nM; H2L5105099 K_i_ = 50 nM; H2L7724589 K_i_ = 311 nM; H2L5226501 maximal inhibition of 59% at 30 μM and NSC47091 inactive, structures shown in Figure 9) [46,47] were docked into each hybrid LPA_1_ receptor model using Autodock Vina. The docking box was centered between the sidechains of W271^6.48^ and S304^7.46^ and extended 40 Å along the long axis of the model and 22 Å along each of the other two axes. Top-ranked poses obtained using searches with exhaustiveness set to 16 were analyzed for consistency with known relative potencies and mutagenesis results.

## 4. Conclusions

**Figure 13:**
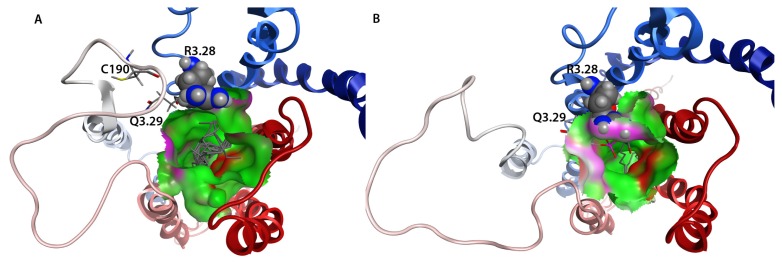
Comparison of ligand binding pockets from a model inconsistent with mutagenesis (panel **A**) and from Figure 11 (panel **B**). LPA_1_ is shown as ribbons shading from blue at the amino terminus to red at the carboxy terminus in all panels. R3.28 is shown as a spacefilling model, with other residues and LPA shown using stick models in all panels.

In conclusion, this study has demonstrated that the two peptide sequences designed by Oakley *et al.*, to form an antiparallel coiled-coil dimer in an aqueous solution, can also self-assemble within a single peptide sequence that included a long linker of 19 amino acids from the LPA_1_ EL2. The coiled-coil motif provided a receptor-mimetic folding constraint for the longest LPA_1_ extracellular loop. This folding constraint made possible atomic-resolution structural studies of the LPA_1_ EL2 in solution, permitting any intrinsic flexibility of the receptor loop sequence to be reflected in the structural data. Inclusion of the coiled-coil is a feasible alternative to performing NMR structural studies of full-length GPCR proteins embedded in membranes or detergents, although with the limitation that structural features dependent on interactions with other loops will not be observed. The NMR data collected for the LPA_1_-CC-EL2 sequence produced a well-converged coiled-coil structure for these designed segments of the overall peptide sequence. In contrast, the native LPA_1_ EL2 loop sequence positioned between these coiled-coil sequences showed convergence only at its *C*-terminal end, which exhibited an outward bend relative to the loop’s position within the full-length LPA_1_ receptor. This lack of structural convergence for the remainder of the loop sequence suggests high intrinsic flexibility under reducing conditions. The majority of structures that were produced during the model generation and refinement process were used in generating hybrid full-length LPA_1_ models without excessive disruption of validated portions of the original model. However, only nine of these hybrid models produced top-ranking docking poses for LPA 18:1 that were consistent with mutagenesis studies demonstrating that R120^3.28^ and E121^3.29^ are essential for LPA 18:1 activity. Figure 13 compares top-down views of a model inconsistent with mutagenesis and that shown in Figure 11. This top view demonstrates that residues in EL2 can influence the relative orientation of the R120^3.28^ sidechain, and therefore the binding mode of LPA identified during docking. Figure 13A shows that C190 in EL2 (corresponding to C36 in the LPA_1_-CC-EL2 sequence shown in Figure 2) forms a hydrogen bond with the R120^3.28^ sidechain in the hybrid model, tipping the hydrogen bond donors up and away from the ligand binding pocket relative to the model shown in Figure 13B. This produces a more hydrophobic pocket, reflected in the docked positions of LPA in this model with the hydrophobic tails positioned near R120^3.28^. It is quite possible that consideration of sidechain flexibility during docking may have allowed the model shown in Figure 13A to produce docked poses more consistent with mutagenesis results. One model was consistent not only with validated LPA interaction sites, but also showed consistency with pharmacological trends of five compounds with varied antagonist activity. Future studies will need to address what pattern of disulfide bond linkages occurs among the six cysteine residues found in EL2 and EL3 of LPA_1_ in order to further refine our understanding of the conformations this sequence is able to explore in the context of the full-length receptor, as the ensemble of structures did not show a natural propensity for specific cysteine residues to be in proximity to each other.

## Figures and Tables

**Table 1:** Statistics from the CNS structure family of LPA_1_-CC-EL2.

Distance Restraints	
No. of total restraints	858
No. of intraresidue (*i* = *j*)	274
No. of interresidue ((*i* − *j*) = 1, (*i* + *j*) = 1)	279
No. of medium (1 < (*i* − *j*) < 5)	150
No. of long ((*i* − *j*) ≥ 5)	12
No. of dihedral-angle constraints (Φ and Ψ)	119
No. of hydrogen bonds	24

E_total_ (kcal/mol)	3149.26 ± 32.19
RMSDs from experimental restraints (CNS defaults)	
Bonds (Å)	0.0079 ± 0.0002
Angles (°)	1.141 ± 0.021
Impropers (°)	1.099 ± 0.032
NOEs	0.119 ± 0.002
Dihedrals	0.677 ± 0.139

Coordinate superimpose	
Backbone RMSD (Å) (2–79)	4.01
Backbone RMSD (Å) (3–30)	0.63
Backbone RMSD (Å) (53–78)	0.58
Backbone RMSD (Å) (3–30 and 53–78)	0.95
